# Confocal Absorbance‐Activated Droplet Sorting (cAADS) for Enzyme Engineering

**DOI:** 10.1002/advs.202505324

**Published:** 2025-08-14

**Authors:** Abdi Mirgissa Kaba, Sébastien Gounel, Thomas Beneyton, Lionel Buisson, Jean‐Christophe Baret, Nicolas Mano

**Affiliations:** ^1^ Centre de Recherche Paul Pascal (CRPP), CNRS UMR 5031 Univ. Bordeaux 115 Avenue du Docteur Schweitzer Pessac 33600 France

**Keywords:** absorbance‐activated droplet screening, bilirubin oxidase, directed evolution, droplet‐based microfluidics, high‐throughput screening

## Abstract

Directed evolution relies on iterative cycles of variant generation, screening, and selection to identify enzyme variants with improved activities. Droplet‐based microfluidics accelerates this process by enabling rapid screening of enzyme variants in water‐in‐oil emulsions acting as picoliter‐scale microcompartments. In fluorescence‐activated droplet sorting (FADS), single *E.coli* cells are screened using a fluorogenic substrate at high throughput (≈2 kHz). However, fluorogenic assays for enzymatic systems are limited, while absorbance‐based detection assays represent a larger spectrum. At the micron scale, light absorption is weak, and scattering induced by droplet interfacial curvature further decreases detection sensitivity. Measurements are therefore performed at a cost of increasing droplet sizes or acquisition times, which limits throughput to <1 kHz. Here, this challenge is addressed with a confocal Absorbance‐Activated Droplet Sorting (cAADS) system. The platform achieves sensitive absorbance measurements at ultrahigh throughput (5.4 kHz) from droplets as small as 10 pL, and sorting of 50 pL droplets at frequencies up to 2.6 kHz. The cAADS methodology is demonstrated by enrichment of active Bilirubin Oxidase (BOD) variants, with a sorting efficiency of 99%. Its versatility and potential for absorbance‐based microfluidic screening in enzyme engineering are also demonstrated using a different enzyme: Glucose Oxidase.

## Graphical abstract

A custom confocal absorbance‐activated droplet sorting (cAADS) platform is presented, which confines illumination and detection to a small area on the “droplet plane”, enabling enhanced sensitivity at ultrahigh throughput. System performances and versatility are demonstrated by screening Bilirubin Oxidase encapsulated in 10 pL droplets at 5.4 kHz, achieving sorting at 2.6 kHz using 50 pL droplets, and by screening Glucose Oxidase.

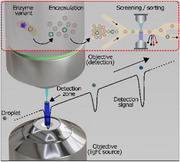

## 1 Introduction

Enzymes play a crucial catalytic role in biological systems to selectively accelerate chemical transformations^[^ ^1^ ^]^ and, as biocatalysts, enzymes are widely used in industrial processes.^[^ ^2^, ^3^ ^]^ Their catalytical properties for electrochemical reactions are appealing for autonomous biosensing and energy conversion in biofuel cells.^[^ ^3^, ^4^, ^5^, ^6^ ^]^ However, the natural performance of most enzymes is often insufficient for current applications: activities of natural enzymes are the result of billion years of evolution, but the optimizations that nature has achieved are not necessarily those required for the use of enzymes in specific physiological, laboratory, or industrial conditions.^[^ ^7^, ^8^ ^]^ Directed Evolution addresses this limitation, by providing a controlled lab‐scale evolution process to optimize enzymes according to the end‐user constraints.^[^ ^9^, ^10^, ^11^, ^12^, ^13^ ^]^ The process consists of three key steps, iterative mutation (i.e., generation of variants), screening, and selecting the best‐performing variant.^[^ ^14^ ^]^ The success of a directed evolution campaign is measured by the ability to find a rare target variant from a substantially large pool of sequences.^[^ ^15^ ^]^ However, the screening and ultimate evolution of the proteins is possible only if the pool of mutant sequences (genotype) retains a direct link to the corresponding enzyme variant (phenotype). The most commonly used screening techniques utilize substrates with a fluorescent or colorimetric readout as a proxy for enzymatic activity. Unfortunately, optical screening using conventional agar and microtiter plates has been a significantly slow process due to the large size of the mutant library, calling for a faster (high‐throughput) alternative.^[^ ^14^, ^16^ ^]^

Miniaturization of the reaction vessel to picoliter (pL)‐volume water‐in‐oil emulsions via microfluidic devices has been shown to be an ideal alternative, allowing a screening ≈1000‐fold faster than microtiter plates.^[^ ^15^ ^]^ In addition to preserving the necessary link between the genotype and phenotype, these surfactant‐stabilized monodisperse droplets are optimal for directed evolution. They offer long‐term stability under various physiochemical conditions, precise control of the reactants, and a reduced reagent consumption, which ultimately reduces the cost of biomolecular analysis.^[^ ^17^ ^]^ Most of the directed evolution achievements in droplet‐based microfluidics have relied on a sensitive laser‐based fluorescence readout known as fluorescence‐activated droplet sorting (FADS), which allows detection and sorting of target variants in the kilohertz range.^[^ ^18^, ^19^, ^20^, ^21^, ^22^ ^]^ In such a process, the enzyme variant is co‐encapsulated with a fluorogenic substrate producing a change in fluorescence intensity based on the activity of the enzyme variant. However, fluorogenic assays are not available for several enzymatic assays.^[^ ^23^ ^]^ In addition, since the fluorophore‐bound substrate is a chemically modified version of the native substrate, the improved enzyme activity may be limited to the modified fluorogenic substrate only.^[^ ^15^ ^]^ Activation of the fluorophore via a coupled assay has been proposed as a solution, at the cost of generating non‐specific side reactions.^[^ ^24^ ^]^ Absorption‐based detection appears as a potential alternative of interest.^[^ ^23^, ^24^, ^25^, ^26^ ^]^ Relying on absorbance detection opens up the opportunity of using a wide range of chromogenic assays and previously inaccessible substrates. One of the first absorbance‐activated droplet sorting (AADS) systems was used for the directed evolution of phenylalanine dehydrogenase and glucose dehydrogenase.^[^ ^24^ ^]^ The study used a microfluidic sorter with two embedded optical fibers facing each other across a microchannel to measure the change in transmittance and then sort the droplets. Nevertheless, the sorting throughput in AADS was lower (300 Hz) compared to sorting rates practically achieved with FADS experiments (typically ≈2 kHz). This lower throughput is due to the use of relatively larger droplets (180 pL), resulting in larger optical path often used in AADS.^[^ ^24^ ^]^

From the fluidic manipulation standpoint, high throughput requires droplets as small as possible. As an example, sorting throughputs in electric fields are larger for 10 pL droplets than 10 nL droplets. On the other hand, from an optics standpoint, absorption scales with the optical path in the sample, which decreases with decreasing droplet sizes. And scattering becomes increasingly important as the interface curvature increases. Solutions to these constraints are currently not satisfactory. They rely on the engineering of microfluidic chips that are either expensive or inconvenient: droplets are squeezed in a microfluidic channel along the direction of illumination to increase the length of the optical path, enhancing sensitivity, but the system can hardly be further downscaled.^[^ ^26^ ^]^ Scattering and refraction artefacts are addressed by integrating collimating lenses into the microfluidic PDMS device.^[^ ^25^ ^]^ A remarkable sorting frequency of 1 kHz has been demonstrated, however, the micro‐lenses made out of PDMS cavities are highly susceptible to deformation, limiting the reliability of the system. Scattering at the aqueous‐oil interface is reduced by matching the refractive index of the oil and the aqueous phases. Screening at 1 kHz with droplets as small as 75 pL has been demonstrated using a formulation based on fluorinated oil with a refractive index matching compound (1,3‐bis(trifluoromethyl)‐5‐bromobenzene).^[^ ^23^ ^]^ Nevertheless, acting on formulations impacts several physicochemical properties, including surface tension, potential leakage of the aqueous phase, and possibly incompatibility with the biological content of the droplets. Background signal reduction has been addressed for example with a lithographic mask with a transparent window aligned to a microchannel.^[^ ^27^ ^]^ However due to the opacity of the mask and its permanent attachment to the chip, the flexibility of the system to optically monitor upstream of the detection zone and the ease with which the detection location can be repositioned are limited, respectively. Relying on such a method, measurements from 50 pL droplets have been demonstrated with a good sorting efficiency at 1.5 kHz (85%). Finally, improving signals can also be done using biological and chemical methods, for example by growing the enzyme‐expressing cells inside the droplet to increase its local concentration.^[^ ^28^ ^]^

There is still an unmet need to optimize the screening capabilities of AADS systems, especially considering FADS as a benchmark. We present here a confocal AADS (cAADS) setup that improves the performance of absorbance‐based droplet detection and sorting capabilities, achieving a screening performance comparable to FADS by reliably detecting signal from a previously unattainable small droplet size. We apply this method to the cell‐based screening of bilirubin oxidase, an enzyme of interest for the elaboration of cathodes for biofuel cells operating under physiological conditions.^[^ ^29^, ^30^, ^31^, ^32^ ^]^

## 2 Results and Discussion

### 2.1 Absorbance Measurement

**Figure 1:**
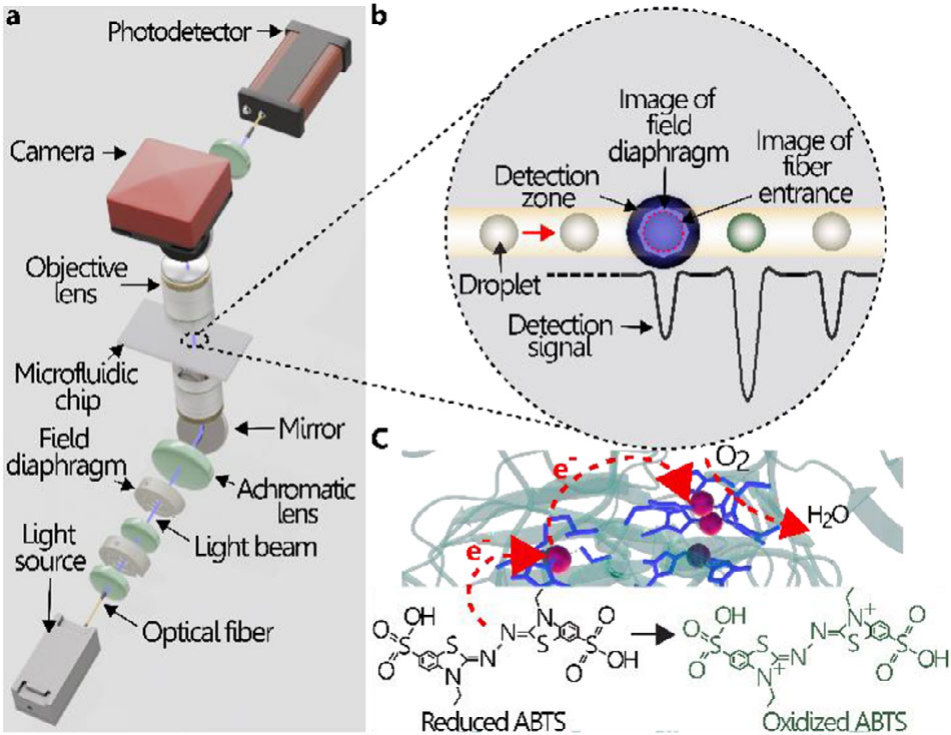
a) Schematic representation of the custom confocal optical setup. Light (455 nm) was passed through a series of optical elements including a collimator, diaphragms, and an achromatic lens before being reflected by a conventional mirror and uniformly illuminated onto the droplet using a condenser (bottom objective). The light was then collected by an objective focused on the “droplet plane” from the top, passed through a tube lens, reflected by a mirror, and coupled to an optical fiber that was connected to a photodetector. b) Inset showing a schematic of active (blue‐green, ABTS encapsulated with an active BOD enzyme) and inactive (beige, ABTS encapsulated with an inactive BOD variant) droplets passing through the detection zone. c) The BOD enzyme accepts electrons from the reduced ABTS substrate at the Type 1 (T1) copper site (single red sphere, left) leading to the formation of a blue‐green product (ABTS^+^) and relays them to the tri‐nuclear cluster (three red spheres, right), where O_2_ reduction takes place. A higher detection signal is obtained from droplets containing the active enzyme.

Our primary goal is to set up an absorbance‐measurement platform capable of accurately measuring the absorbance in droplets of <100 pL volume at high throughput (>1 kHz). Here, absorbance is measured using a custom‐built confocal microscope (**Figure** **1**a; Figure S1, Supporting Information), designed for absorbance‐based detection of the oxidized form of 2,2’‐azino‐bis(3‐ethylbenzothiazoline‐6‐sulfonic acid) (ABTS).

The oxidation is catalyzed by bilirubin oxidase (BOD) from *Bacillus pumilus*, which will be used as a model enzyme for screening applications. The confocal imaging configuration enables selective and precise absorbance detection of droplets within a microchannel by ensuring that both the illumination and detection optics are spatially conjugated and focused on the same diffraction‐limited spot.^[^ ^33^ ^]^ This configuration effectively rejects out‐of‐focus scattered light, especially at high numerical aperture (NA), that would otherwise contribute to increased background signal and reduced contrast. Briefly, light is focused at an aperture diaphragm and then at the rear focal plane of a condenser (50× objective lens) after passing through a tube lens (Figure S1, Supporting Information). The objective has a numerical aperture (NA) that matches the NA of the same transmitted‐light‐collecting objective (field of view = 0.46 mm and depth of field = 1.8 µm) at the top. This arrangement significantly reduces the collection of light from other planes and from refraction at the droplet/oil interface. A field diaphragm placed between a collimating lens and the tube lens is also conjugated to the sample and image planes. This allows us to easily adjust the position and size of the field diaphragm to match the size of the droplet passing through the PDMS microchannel, which is aligned along the imaging axis and is focused using a precision XYZ stage. After passing through the droplet and an imaging lens, the light is then split to a camera and focused through an aspheric lens to a detection optical fiber. The entrance of the fiber is thus conjugated with the droplet, which is initially confirmed by superimposing the image of both on the image of the field diaphragm. The output signal from a photodetector connected to the detection optical fiber is measured by a multifunction data acquisition hardware controlled by LabVIEW.

### 2.2 Calibration of Optical Setup

**Figure 2:**
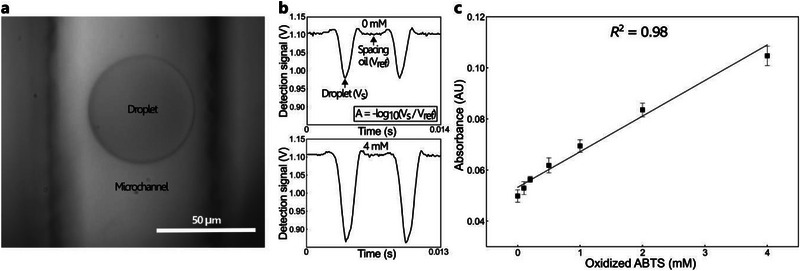
a) Micrograph showing a droplet (100 pL) passing through the detection zone of a microchannel in the analysis module. The droplet contains oxidized ABTS (4 mm). b) Time course of the detection signal for light transmitted at 455 nm through a series of droplet populations with concentrations of 0 mm (top) and 4 mm (bottom) oxidized ABTS. While the signal from the spacing oil remains constant (baseline), it decreases as the droplets pass through the detection zone with a peak minima at the center of each droplet. As expected, the detected light intensity decreases with increasing substrate concentration. The absorbance (*A*) is calculated from the logarithmic relationship between the droplet voltage (*V_s_*)* *and the reference voltage (*V_ref_*) as shown in the inset. c) Calibration plot depicting an increase in absorbance for an increase in oxidized ABTS concentration from 0 to 4 mm. The data is shown as mean±SD of 1000 data points at each concentration.

We first analyse the optical signal from a single population of 100 pL droplets containing a pure BOD enzyme (0.097 mg mL^−1^; Section S2, Supporting Information) and ABTS substrate (4 mm), and compare it to a negative control (0 mm ABTS). The substrate is first co‐compartmentalized with a pure BOD enzyme using a single dropmaker module (Figure S3a, Supporting Information), and the droplets are collected off‐chip in a glass vial. After complete oxidation of the ABTS, the droplets are passed through the detection zone of the analysis module (**Figure** **2**a; Figure S3c, Supporting Information). Droplets are detected as downward peaks in transmittance; a negligible scatter peak at the interface, and a difference between the 0 and 4 mm is observed (Figure 2b). The Absorbance (*A*) is defined as *A* = −*log*_10_(*V_s_*/*V_ref_*), where *V_s_* is the peak signal voltage at a given concentration and *V_ref_* is the baseline voltage from the spacing oil. The sensitivity is characterized by measuring the absorbance of 100 pL droplets with ABTS concentration ranging from 0 to 4 mm. Absorbance is measured after a 10‐min incubation at room temperature (25 °C; see Section S4, Supporting Information). The data are averaged over 1000 drops at each concentration. The absorbance is shown to increase with ABTS concentration from 0.049 ± 0.002 at 0 mm to 0.105 ± 0.004 at 4 mm with a low standard deviation among droplets using a standard chip fabrication and the confocal measurement, demonstrating that we can reliably detect ABTS concentration as low as 100 µm in a 100 pL droplet (Figure 2c).

### 2.3 Dynamic Range and Droplet‐Size Detection Limit

**Figure 3:**
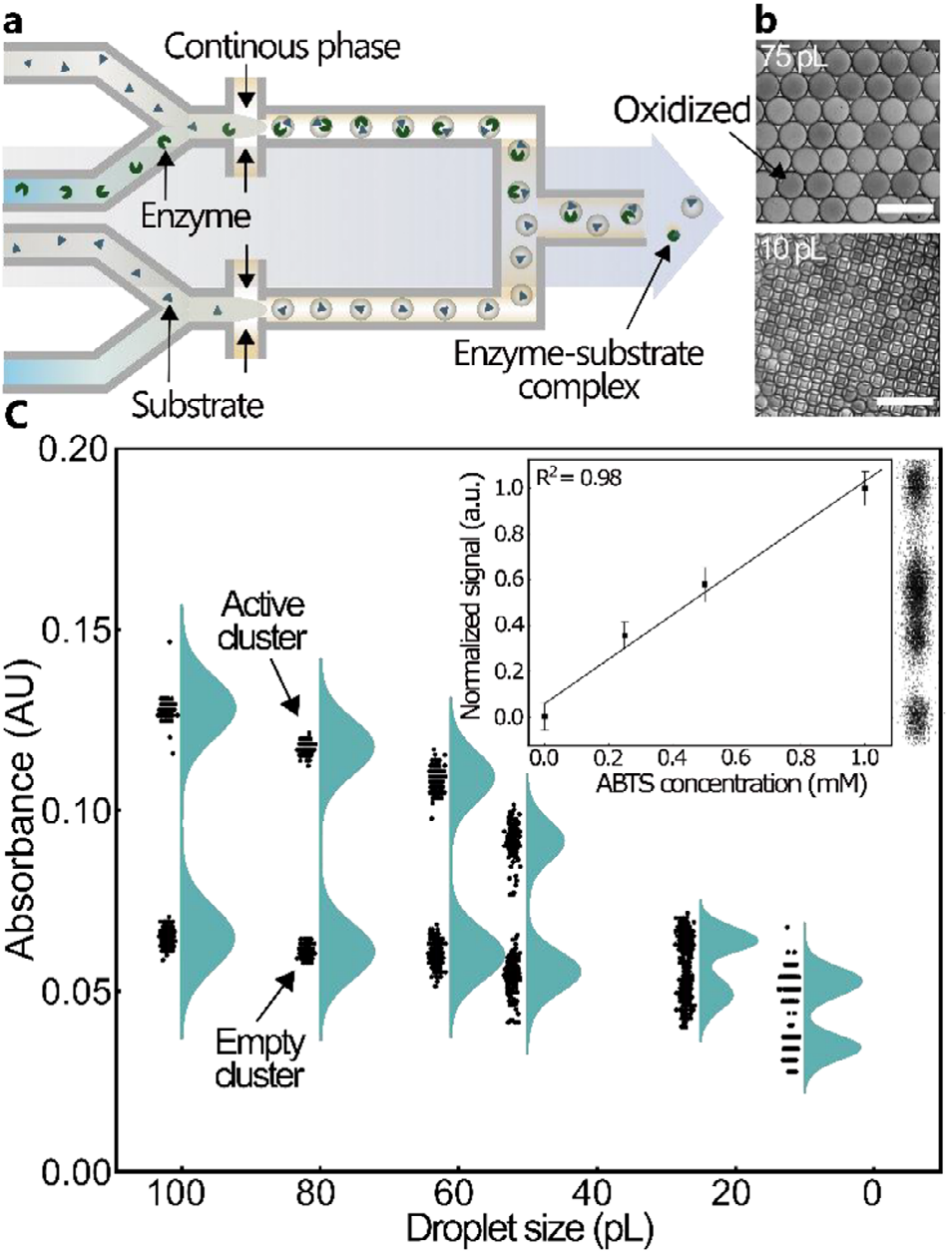
a) Schematic of the dual dropmaker module used to generate a droplet population comprising active (BOD enzyme and ABTS) and empty (ABTS only) droplets. b) Micrograph showing the clear colorimetric difference between the active (oxidized) and empty clusters of 75 pL and 10 pL droplets. c) Violin‐scatter plot generated from the absorbance measured using the cAADS setup shows an accurate discrimination of the two population clusters for a droplet size ranging from 100 to 10 pL. Although sensitivity decreases with decreasing droplet size due to a shorter optical path length, discrimination was successful even for the smallest droplet size. An inset calibration plot, generated using a population of the smallest droplet (10 pL), corroborates this further by illustrating an increase in the detection signal upon increasing oxidized ABTS concentration from 0 to 1 mm. The means and standard deviations were determined by fitting a histogram (Figure S5, Supporting Information) generated from 82 000 droplets to a sum of four Gaussian components. The inset scatter plot shows the distribution of the data. Scale bar: 100 µm.

We further measure the absorbance of droplets of decreasing sizes (10–100 pL). Using a dual dropmaker (Figure S3b, Supporting Information), we produce in parallel droplets containing 10 mm of ABTS co‐compartmentalized with the BOD enzyme and droplets solely containing 10 mm of ABTS (as a negative control; **Figure** **3**a). The Flow conditions used to generate the droplets are indicated in Table S1 (Supporting Information). After a 10‐min incubation at room temperature to ensure completion of the reaction for the entire population within the collection vial, the droplets are reloaded into the analysis module. The droplets containing ABTS and the enzyme undergo a chromogenic change due to the oxidation of ABTS, while the negative control shows no oxidation, as revealed by the contrast difference in microscopic imaging (Figure 3b). Analysis of the signal from 500 droplets for each droplet size shows a clear discrimination between the oxidized (active cluster) and the negative control (empty cluster), as shown on the violin‐scatter plot of Figure 3c. We notice a reduction in absorbance with decreasing droplet size as expected from the reduction in optical path length. Here, we obtain a clear difference between the positive and negative controls down to volumes of 10 pL without additional additives (e.g., gold nanoparticles^[^ ^34^ ^]^), which is five times smaller than the smallest volume reported to date (Figure 3c).^[^ ^27^ ^]^ This experiment then demonstrates the ability to discriminate between the presence or absence of BOD enzymes in droplets down to a volume of 10 pL. The sensitive discrimination capability was further demonstrated by measuring the signal from a population of 10 pL droplets with an ABTS concentration ranging from 0 to 1 mm (Figure 3c, inset) using a cAADS module (Figure S3d, Supporting Information). The average intensity increased from 2.328 ± 0.002 V (normalized mean = 0) at 0 mm to 2.368 ± 0.003 V (normalized mean = 1) at 1 mm using the confocal measurements. This indicates accurate detection of the ABTS concentration down to 250 µm in 10 pL droplets. The 2.5× increase in LOD is consistent with the approximately 2.15× reduction in optical path length from 100 pL droplets (Figure 2c).

To highlight the versatility of our approach to other enzymatic systems, we used a multistep enzyme reaction with glucose oxidase (GOx), a flavin adenine dinucleotide based enzyme,^[^ ^35^ ^]^ and horseradish peroxidase (HRP). We produce in parallel 50 pL droplets containing 10 mm of ABTS and 3 mm of glucose, co‐compartmentalized with the GOx and HRP enzymes, and a negative control with droplets containing the same enzymes and chromogenic substrate, but without glucose. Upon mixture, the GOx catalyzes the oxidation of glucose and produces hydrogen peroxide, which is subsequently used by HRP to oxidize ABTS. Following measurement using the cAADS platform, analysis of the signal shows a clear discrimination between the oxidized (2.300 ± 0.004 V) and the negative cluster (2.280 ± 0.002 V), as depicted on the violin‐scatter plot in Figure S4 (Supporting Information).

### 2.4 Sorting Efficiency

**Figure 4:**
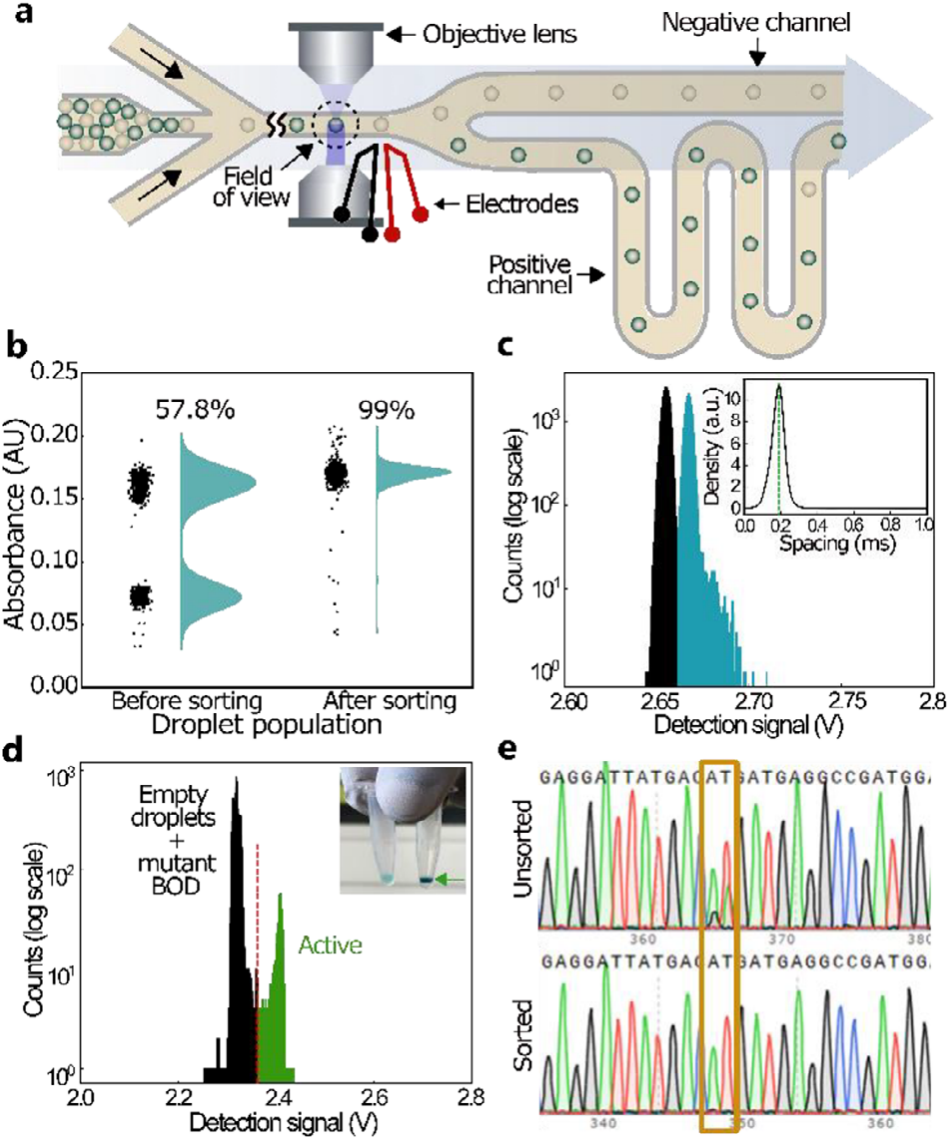
a) Schematic of the cAADS module where incubated droplets are reloaded and sorted based on a defined threshold. A non‐uniform AC electric field applied across the electrodes deflects active droplets into the positive channel. Due to the lower hydraulic resistance of the negative channel, droplets flow into the negative arm when the electric field is not applied. b) Violin‐scatter plot showing the change in the percentage of active droplet population before and after sorting using purified BOD enzyme encapsulated with ABTS. A total of 1000 events were measured. c) 1D histogram depicting the signal from a population of 10 pL droplets containing 10 mm ABTS, both with (light blue) and without (black) BOD enzyme. The inset density plot shows the distribution of the spacing between each measurement (i.e., droplet), with a peak (green dotted line) at 0.187 ms, confirming accurate measurement at 5344 Hz. d) 1D histogram showing the signal from inactive/empty (black) and active (green) droplets after encapsulation (50 pL) and incubation (80 °C, 30 min) of BOD‐expressing *E.coli* with ABTS. Droplets with a signal above the systematically placed threshold (red dotted line) are sorted into the positive channel at frequencies up to 2.6 kHz (Figure S9, Supporting Information) and collected for subsequent analysis. The inset picture shows the clear difference in color between the unsorted total droplet population containing only ≈4.5% active droplets (left) and active (right, green arrow) droplet population after sorting. e) Sanger sequencing of DNA amplified from unsorted (top) and sorted (bottom) droplet populations. The orange box highlights nucleotide peaks corresponding to both the mutant and wild‐type BOD in the unsorted droplet population, whereas peaks only from the wild type are observed in the sorted droplet population.

After demonstrating the ability to discriminate the presence of an enzyme in the droplets, we demonstrate the sorting capability of our cAADS platform. Two populations of monodisperse 50 pL droplets were generated simultaneously using a dual dropmaker. As before, one population contains a pure BOD enzyme and ABTS substrate, while the other contains only ABTS. After droplet formation, the emulsions were incubated at room temperature for 10 min. The droplets were then injected into the cAADS module for sorting, as described previously.^[^ ^18^, ^20^ ^]^ To benchmark the sorting efficiency against FADS, we first optimized the sorting conditions (i.e., high‐voltage AC and oil/sample pumping flow rates) for this droplet size on a separate FADS setup^[^ ^36^ ^]^ with a lower magnification lens (Plan 20×, Olympus) allowing imaging of the downstream. The same sorting module and optimized conditions (Table S2, Supporting Information) were then applied to sorting experiments on the cAADS platform, following a brief empirical investigation to determine the optimal detection zone on the module that guarantees reliable sorting (**Figure** **4**a). Sorting efficiency is calculated as the ratio of active droplets to the total number of droplets (1000) sorted into the positive channel. The FADS showed a 100% sorting efficiency up to a frequency of 2.5 kHz. We also conducted the cAADS sorting tests at similar frequencies. We measured the percentage of positive and negative droplets before and after sorting (Figure 4b). This percentage of active droplets increased from 58% before sorting to 99% after sorting at a conservatively maintained frequency of 2 kHz. The sorting rate of our setup was found to be two to eight times higher than previously reported sorting frequencies of AADS (0.30 kHz,^[^ ^24^ ^]^ 1 kHz,^[^ ^23^ ^]^ 1.5 kHz;^[^ ^27^ ^]^ Figure S6, Supporting Information). We therefore demonstrate that cAADS can function in regimes comparable to those of FADS. To further explore the detection and sorting capabilities of the cAADS platform at even higher frequencies, we prepared two populations of monodisperse 10 pL droplets using a dual dropmaker (Figure S3b, Supporting Information). The formulation and incubation conditions were the same as for the 50 pL droplets. We used a cAADS module with an additional spacing‐oil channel (Figure S3d, Supporting Information) to optimize the sorting conditions for the 10 pL droplets using the aforementioned FADS setup. Under the experimentally optimized sorting conditions (Table S2, Supporting Information), the FADS showed a 100% sorting efficiency up to a frequency of 5.4 kHz (Figure S7 and Movie S1, Supporting Information). The same sorting module and optimized conditions were later used for discrimination and sorting on the cAADS platform. Similarly, accurate screening and discrimination was achieved at frequencies up to 5.4 kHz (Figure 4c; Figure S8, Supporting Information), 3.6× higher than the highest previously reported absorbance‐based droplet screening (Figure S6, Supporting Information).^[^ ^27^ ^]^ However, the inability to visualize the downstream region makes it difficult to identify the exact detection zone required for reliable sorting. This issue is particularly evident at ultrahigh frequency of ≈5 kHz and with small 10 pL droplets, as the droplets travel more rapidly (≈0.85 µm/µs) through the channel. Even with a nominal zero delay setting, the impact of inherent system and electronic delays is exacerbated under these conditions, reducing the time available for accurate actuation of the target droplet. Nevertheless, reliable absorbance measurement of 10 pL droplets, and separately, demonstrated sorting above 5 kHz on a setup with downstream imaging (FADS) suggests that the cAADS platform holds potential for similar ultrahigh‐throughput sorting. This could be achieved by incorporating a supporting imaging system (e.g., an additional objective lens and high‐speed camera downstream of the detection zone).

### 2.5 Enrichment of a Variant Expressing Active BOD

The previous experiments were performed using a purified enzyme, which is not directly applicable to the screening of enzyme libraries. To evaluate the performance of a droplet‐based microfluidic enzyme screening platform, an enrichment based on cell expression of enzymes is more appropriate.^[^ ^18^, ^24^ ^]^ We then demonstrate the separation of bacteria expressing BOD from mixed populations.

We first co‐encapsulate bacterial strain expressing an active wild‐type or inactive mutant enzyme variant (Figure S2, Supporting Information) along with a chromogenic substrate. Here, an *E.coli* strain expressing an active BOD gene and another expressing an inactive variant (M502E) were compartmentalized with ABTS in 50 pL droplets using our dual dropmaker module. During encapsulation, the distribution of cells between droplets followed a Poisson distribution.^[^ ^37^, ^38^ ^]^ Co‐encapsulation of two or more cells in a single droplet was avoided by diluting the cell suspensions to 0.1 cells per 50 pL (λ).^[^ ^19^, ^39^ ^]^ This means that 9% of the droplet population from each unit of the dual dropmaker module contains a single cell, while ≈90.9% are empty, and <1% carry more than one cell. Thus, only ≈4.5% of the total collected droplet population contains the active enzyme. The droplets were then incubated off‐chip at 80 °C for 30 min to ensure cell lysis. The absorbance signal is measured by reloading the droplets into the cAADS module (Figure 4a; Figure S3e, Supporting Information). The emulsions showed two populations in absorbance signal. As expected, the mutant variant is indistinguishable from the empty droplets, as observed in the 1D histogram in Figure 4d.

Using a droplet‐sorting threshold based on the absorbance signal,^[^ ^19^ ^]^ the active droplet population was sorted at 2 kHz for 6 min (i.e., ≈720 000 screened droplets and ≈25 000 collected droplets) and transferred into a low DNA‐binding microcentrifuge tube. The same number of droplets were also collected in a separate microcentrifuge tube without activating the electric field (i.e., unsorted population). The DNA (i.e., plasmid containing the open reading frame of the enzyme) was then recovered, purified, and concentrated before being amplified by two PCR steps (Figure S10, Supporting Information) to obtain a sufficient DNA concentration for Sanger sequencing (see Section S12, Supporting Information). The amplified DNA insert was then sequenced. The sequencing chromatogram from the unsorted droplet population (Figure 4e, top) clearly shows overlapping peaks (orange box) corresponding to both the mutant and wild‐type nucleotides, while the chromatogram from the sorted population (Figure 4e, bottom) shows only wild‐type nucleotide peaks, indicating the absence of sequences from the mutant variant.

We therefore demonstrate here that the cAADS platform has performances compatible with ultrahigh‐throughput screening applications down to a single cell resolution. We have demonstrated detection from droplets as small as 10 pL and a successful sorting frequency of 2 kHz (confirmed via sequencing after enrichment), which puts our system on par with FADS, the gold standard for microfluidic directed‐evolution experiments.^[^ ^18^ ^]^

To summarize and discuss the key features of our approach, we want to highlight that the microfluidic system developed here does not require any modifications compared to standard systems used for AADS, such as fluidic constrictions,^[^ ^26^ ^]^ insertion of optical fibers,^[^ ^23^, ^24^, ^26^ ^]^ microfabrication of lenses^[^ ^25^ ^]^ or microfabricated masking areas,^[^ ^27^ ^]^ which simplifies the microfabrication process. It does not require the use of additives in the oil phase (e.g., refractive index matching compound to minimize scattering^[^ ^23^ ^]^) or in the aqueous phase (e.g., gold nanoparticles to enhance signals^[^ ^34^ ^]^) ensuring the biocompatibility of the formulations for biological applications. It also does not require the growth of cells to enhance enzyme activity,^[^ ^28^ ^]^ which simplifies the microfluidic workflow. In contrast, the sensitivity of our system results from the engineering of an optical system in a confocal mode, thoroughly focusing the imaging objective lens on a plane where the field diaphragm, droplet, and detection‐fiber entrance are aligned, providing accurate absorbance measurements with minimal scatter interference at ultrahigh throughput.

## 3 Conclusion

We propose here a confocal optical platform usable to measure absorbance in droplet‐based microfluidics. We illustrate this approach by measuring oxidized ABTS, which serves as a reporter for bilirubin oxidase activity. We show that our approach significantly improves the droplet detection limit, allowing the detection of enzyme activity in smaller volumes (down to 10 pL). The gain in sensitivity enables measurements at the single cell level in 50 pL droplets with a significant increase in throughput (i.e., two to eight times higher compared to previous works). We show the ultrahigh‐throughput sorting of distinct variants at a frequency of up to 2.6 kHz in 50 pL droplets, comparable to FADS. Moreover, we demonstrate an ultrahigh throughput screening capability of 5.4 kHz using 10 pL droplets, 3.6× faster than the fastest reported absorbance‐based droplet screening.^[^ ^27^ ^]^ Versatility of the platform is also shown by screening droplets with chromogenic changes from multistep enzymatic reactions catalysed by glucose oxidase and horseradish peroxidase. Our approach solely requires the engineering of optical detection and no modifications to the microfluidic hardware (the microfluidic chips) and formulations (the oil and surfactant mixture). The combination of high sorting frequency, ease of fabrication, and seamless maneuverability makes this platform a powerful and versatile tool for absorbance‐based droplet microfluidic directed evolution and other high‐throughput biochemical applications.

## Conflict of Interest

The authors declare no conflict of interest.

## Supporting information



Supporting Information

## Data Availability

The data that support the findings of this study are available from the corresponding author upon reasonable request.
