# Supplementary material for: Confocal Absorbance‐Activated Droplet Sorting (cAADS) for Enzyme Engineering
Source: Adv Sci (Weinh). 2025 Aug 14;12(41):e05324. doi: 10.1002/advs.202505324 (PMC12591201; doi:10.1002/advs.202505324)
Supplement: Supplementary file 1 — Supporting Information [file ADVS-12-e05324-s001.pdf]

Supporting Information for

Confocal Absorbance-Activated Droplet Sorting (cAADS) for Enzyme Engineering

Abdi Mirgissa Kaba, Sébastien Gounel, Thomas Beneyton, Lionel Buisson,

Jean-Christophe Baret*, and Nicolas Mano*

Centre de Recherche Paul Pascal (CRPP), CNRS UMR 5031, Univ. Bordeaux, Pessac, France

* Corresponding author

Email address: jean-christophe.baret@u-bordeaux.fr; nicolas.mano@crpp.cnrs.fr

Table of Contents

S.1 Confocal optical setup S-2
S.2 Enzyme preparation and purification S-4
S.3 Microfluidic devices and droplet making S-5
S.4 BOD activity in droplets S-8
S.5 Functional scope of the system S-9
S.6 Detection limit at 10 pL S-10
S.7 Experimental determination of sorting condition S-11
S.8 Comparative performance analysis of cAADS S-12
S.9 Ultrahigh throughput fluorescence-activated droplet sorting (FADS) S-13
S.10 Ultrahigh-throughput screening on cAADS platform S-15
S.11 Enrichment of active BOD S-16
S.12 Plasmid DNA recovery, concentration, amplification, and sequencing S-17
References S-18

S.1 Confocal optical setup

Absorbance is measured using a custom-built confocal microscope. Köhler illumination is achieved using a fiber-coupled LED (455 nm, M455F3, Thorlabs) conjugated to the aperture diaphragm (SM1D12C) via two lenses (L1: AC254-030-A-ML and L2: AC254-035-A-ML, Thorlabs) as shown in Figure S1. The light is then collimated by lens L3 and passed through a field diaphragm before being focused by a tube lens L4 (AC508-180-A-ML) which directs it to the back focal plane of a condenser (objective lens, LD Epiplan 50×/0.50, Zeiss). This objective lens has a numerical aperture (NA) that perfectly matches the NA of the top objective lens used for light collection. These objectives were selected for their long working distance (WD = 6.9 mm) and their ability to meet the system's optical requirements. The field depth is 1.8 μm. The field diaphragm is conjugated to the sample and image planes. This setup allows precise adjustment of the position and size of the field diaphragm to fit droplets flowing through the PDMS microchannel. The microfluidic chip is mounted on a precision XYZ stage, enabling accurate alignment and focus of the channel along the imaging axis. The light collected by the top objective is split using a beam splitter (BS016, Thorlabs). Part of the light is directed to a camera (UI-3240CP, Thorlabs) through an imaging lens L5 with a focal length of 150 mm (AC254-150-A-ML). The other part is directed to a detection optical fiber via an aspheric lens L6 (C230TM-A, Thorlabs). The entrance of the optical fiber is aligned with the droplet by superimposing its image onto the images of the field diaphragm and droplet. The fiber is connected to a photodetector (PDA36A-EC, Thorlabs), which measures the transmitted light. For screening, the signal from the detector is sent to a field-programmable gate array (FPGA, PCI-7831R, National Instruments) running a LabVIEW program. Droplets are identified based on the peaks in their transmittance signals and are dielectrophoretically sorted according to a user-defined threshold.

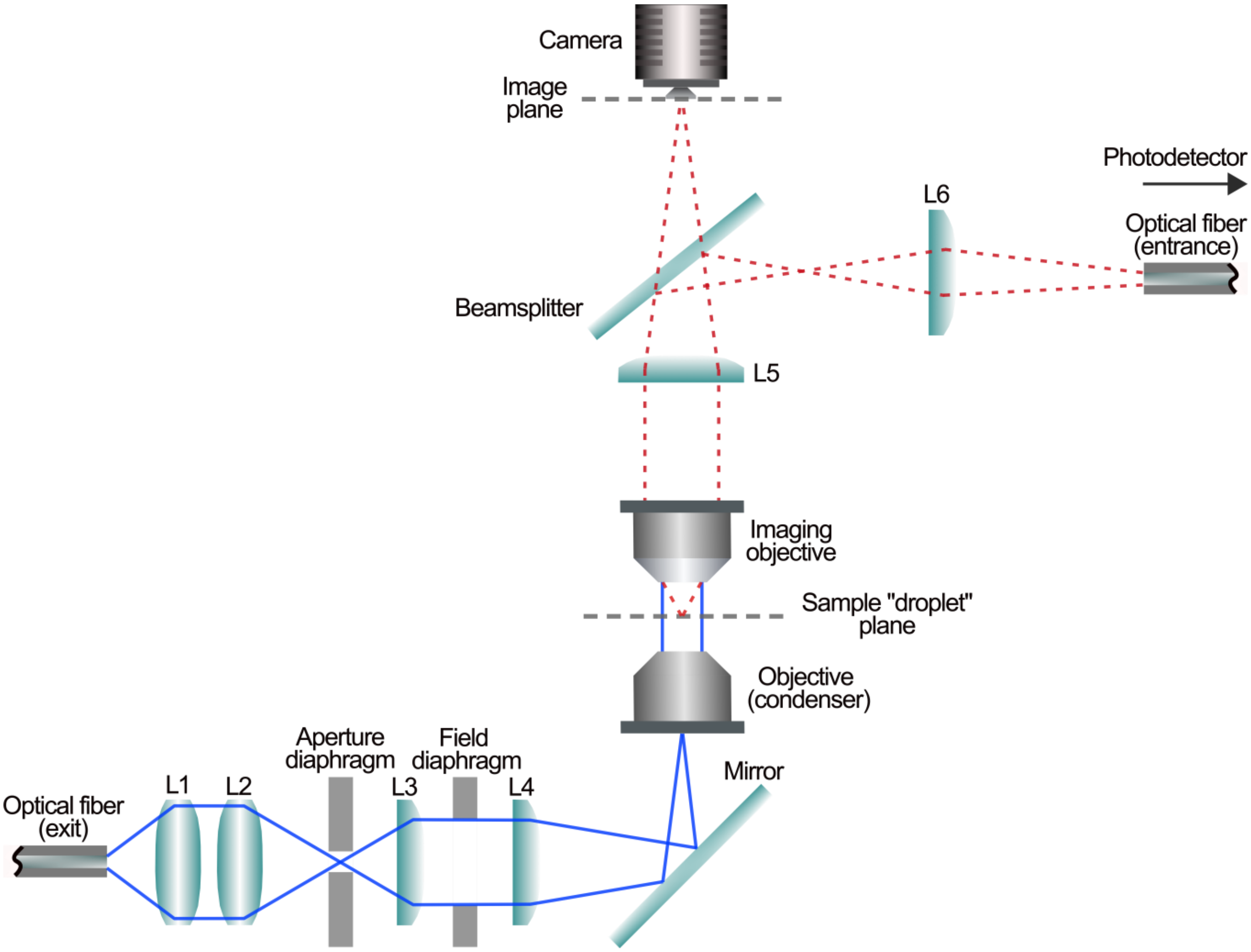

Figure S1. Schematic of the optical layout. Blue solid lines depict the illumination path while the red dotted lines show the sample imaging path.

S.2 Enzyme preparation and purification

The coding sequence of the BOD from *B. pumilus* was integrated into a pet21a under the control of a T7 promoter as previously described.[1] The M502E mutation was performed using the "quick change mutagenesis" kit (Agilent) and the following primers:

Forward: atccacgaggaccacgacgagatggccgccttcaacgtg

Reverse: cacgttgaaggcggccatctcgtcgtggtcctcgtggat.

The nucleotide sequence of the wild-type and mutant enzymes is shown in Figure S2. The production of these enzymes in E. coli Origami B (DE3) strains has been described previously.[2] After production, for experiments conducted in bacteria, 2 mL aliquots are centrifuged for 3 minutes at 16,000 g and 4 °C, and the pellets are stored at -20 °C. For experiments conducted with purified enzymes, the enzymes were purified using HisprepFF 16/10 columns as previously described[2] and stored at -80 °C. The specific activity was determined spectrophotometrically at 37 °C by following the oxidation of 1 mM of ABTS at 420 nm (molar absorption coefficient $\varepsilon_{420\,nm} = 36\ mM^{-1}\ cm^{-1}$) in a 0.1 M Mcllvaine's citrate-phosphate buffer at pH 4. One unit was defined as the amount of enzymes that oxidized 1 µmol of substrate per minute. Glucose Oxidase from *A. niger* was purified as previously reported.[3]

Wild type

```
atgaacctagaaaaatttgttgacgagctgccaattccagaagttgcggagcccgtcaaaaagaacccaagacaaacatattatgaaatcgctatggaggaggtatt
tctaaaagttcatagagacctgcccccaaccaaactatggacctataatggcagtttgcctggtccaaccattcatgcaaatcgaaatgaaaaagttaaagtgaaat
ggatgaacaaattgccacttaaacatttctaccggtcgatcacacgattcacgaaggccatcatgatgaaccagaagtcaagaccgtcgttcatttacatggcggc
gtcacaccagcaagcagtgacggctatccagaggcttggttttcacgagactttgaagcaaccggccccttctttgaacgggaagtgtacgaatacccaaatcatca
gcaagcctgcacattgtggtatcacgatcatgcgatggcattgacacgattaaatgtgtacgccggattagctggattttatttgatctcagatgcgtttgaaaaat
cactagaattaccgaaggatgagtatgatattccgctaatgatcatggaccgtacgtttcaagaggatggcgcgctgttttatccaagcagaccaaacaacacgcca
gaagacagtgatctaccagatccgtctatcgtgcccttcttttgcggagaaaccattttggtcaatggaaaagtatggccatatttagaagtagaaccacgaaaata
ccgttttcgtattttaaatgcgtctaatacaagaacttacgagctgcatctagacaacgatgcgacgattttgcaaattggatctgatggcggctttttaccaagac
ctgttcaccatcaatcctttaccattgctcctgctgaacgatttgatgtgatcattgattttcagcttacgaaaacaaaacgatcacccttaaaaataaagctggc
tgcggacaggaagtaaatcctgaaacagatgccaacatcatgcaatttaaagtcacacgtccactaaaagggagagcacctaaaacattacggcctatattcaaacc
gcttccaccacttcgacctagtcgcgctgatcaagagcgtacgctcactcttactggtacacaggataaatacggtcgccctattttattgcttgataaccattttt
ggaatgaccctgtcacggaaaatcctcggcttggcagtgtagaggtttggtccatcgtcaatccaacaaggggcacacatcccattcatttacatcttgttcaattt
agggtgatagacagaagaccatttgatacagaggtctatcaatcgacaggggacattgtgtatacaggaccgaacgaagcccctcctttacatgaacaaggctacaa
ggacaccattcaagcgcatgccggtgaagtcattcgaatcattgctcgctttgttccatacagcggcaggtacgtgtggcattgtcatatattagagcacgaggatt
atgacatgatgaggccgatggatattatttag
```

Mutant

```
..gacgagatg..
```

Figure S2. Nucleotide sequence of the wild-type and mutant plasmid with a point mutation from the bold green to the bold black colored codon.

S.3 Microfluidic devices and droplet making

Microfluidic devices were prepared from poly-(dimethysiloxane) (PDMS) using standard soft-lithography techniques. The microfluidic features were designed using AutoCAD (Autodesk), followed by the fabrication of the transparent photomask (CAD/Art Services, Inc., SELBA S. A.). A negative photoresist (SU-8 3050, Microchem Corp.) was spin-coated on a silicon wafer (Si-Mat Silicon Materials) and exposed to UV light (MJB4 contact mask aligner, SUSS MicroTec) through the photomask. After exposure, a SU-8 developer (microChem) was used to develop the molds. Subsequently, a PDMS-curing agent mixture (10% w/w, Sylgard 184 silicone elastomer kit) was degassed for approximately 5 minutes and poured over the molds. After curing at 65 °C overnight, the PDMS was peeled off from the molds and the input and output ports were punched using a 0.75-mm biopsy punch. The PDMS slabs and 76 × 26 × 1.2-mm glass slides (Corning) were rinsed with isopropanol and dried using pressurized nitrogen gas before exposing both to oxygen-plasma (PICO, Diener electronic) and pressing them together. The microfluidic channels were made hydrophobic by injecting fluoro-silane (Aquapel, PPG Industries). The channels were flushed with a pressurized argon gas pre- and post-Aquapel injection. The sorting chips were prepared by bonding the PDMS slab to the non-conductive side of an indium tin oxide glass (ITO, 76 × 26 × 1.1 mm, Delta Technologies). The microfluidic device was heated to 120 °C using a hot plate and the channels dedicated to the electrodes were filled with a low-melting point solder (Indalloy 19, Indium Corporation). Finally, electrical wires (Radiospare) were attached to the solder electrodes for electrical connections.

Three different microfluidic modules with a channel depth of 25 μm were used in this study. First, a dropmaker module with a nozzle width of 40 μm (Figure S3a, oil and two aqueous inlets) was used to make a single population of 100-pL droplets by flow-focusing of the aqueous stream with two streams of fluorinated oil containing 1% (w/w) of surfactant. A dual dropmaker with two dropmaker modules in parallel (Figure S3b) having a nozzle width identical to the previous module, was used to simultaneously generate two populations of 60 pL, 80 pL, and 100 pL droplets. A similar module with a nozzle width of 20 μm was used to make 10-pL, 25-pL, and 50-pL droplets. The Flow conditions used to generate the droplets are indicated in the table below (Table S1). The generated droplets were passed off-chip through a PTFE tubing and collected in a glass vial. To study the sensitivity, limit of detection (LOD) and droplet-size detection limit of the optical setup, a simple analysis module (Figure S3c) with a straight 80-μm-wide channel, two inlets (spacing oil and emulsion) and a single outlet was used. For screening and sorting 10 pL droplets, a cAADS module (Figure S3d) with three inlets (two spacing oil and emuslsion) and two collection outlets (sorted and waste) was used. For sorting 50 pL droplets, a cAADS module (Figure S3e), composed of two inlets (spacing oil and emulsion) and two collection outlets) was used.

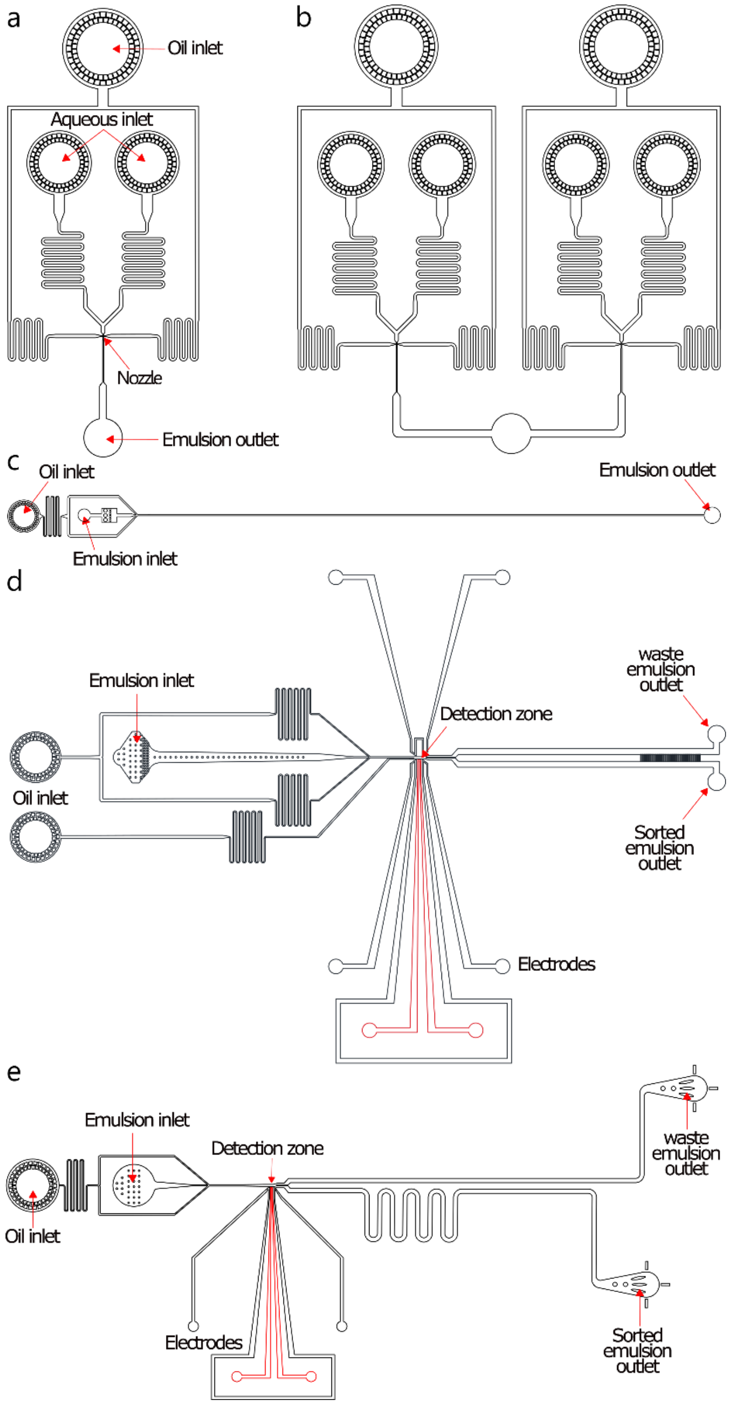

Figure S3. Design of the single- and dual-dropmaker modules: (a) a single-dropmaker module with three inlets (two aqueous and one oil) and one outlet, used to make a single population of emulsion, and (b) a dual-dropmaker module with six inlets (four aqueous and two oil) and two outlets, used to simultaneously generate two populations of emulsions that are collected via a single outlet. (c) Analysis module with one emulsion and one oil inlet, and one outlet. (d) cAADS module with one emulsion and two oil inlets, and one outlet each for sorted and waste enulsions. (e) cAADS module with one emulsion and one oil inlet, and one outlet for sorted and waste emulsions each.

Table S1. Pumping condition for different droplet size

No	Droplet size (pL)	Flow rate Q (μL/hr)		
		**Enzyme**	**Substrate**	**Oil + Surfactant ***
**1**	10	70	70	1070
**2**	25	100	100	645
**3**	50	100	100	150
**4**	60	100	100	260
**5**	80	100	100	160
**6**	100	100	100	120

*Average

S.4 BOD activity in droplets

To investigate the limit of detection of the optical setup and the linearity of the measured absorbance for a range of substrate concentrations (ABTS, 0 – 4 mM) compartmentalized with a pure BOD enzyme (0.097 mg/mL), 100-pL droplets generated using the single dropmaker module were used. After completion of the droplet making, the emulsion in the glass vial was incubated for 10 min at room temperature (25 °C) before being reinjected into the analysis module. Droplet-size detection limit of the setup was also examined using the same module after simultaneously co-encapsulating (dual dropmaker) 10 mM ABTS with and without pure BOD enzyme in droplets with size ranging from 10 to 100 pL, followed by incubation (10 min, 25 °C). For enrichment analysis, a library sorting experiment was mimicked to accurately sort droplets containing wild-type BOD from a large population of droplets containing either an inactive variant (M502E) and the substrate or the substrate only. Thus, *Escherichia coli* cell suspensions expressing each strain were diluted to 0.1 cells / 50 pL (to reduce co-encapsulation)[4] and encapsulated using a dual dropmaker. Subsequently, the collected emulsion was then incubated off-chip for 30 min at 80 °C and reinjected into the cAADS module for sorting.

S.5 Functional scope of the system

A multistep enzyme reaction based on glucose oxidase (GOx) and horseradish peroxidase (HRP) enzymes was used to study the applicability of the cAADS platform to enzymes other than bilirubin oxidase (BOD). A double dropmaker comprising one unit with glucose and ABTS in one channel and the enzymes in the other, and the second unit with the same arrangement but without glucose (negative control) was used to make monodisperse 50-pL droplets containing 3 mM glucose, 10 mM ABTS, 0.31 mg/ml HRP, and 5 mg/ml GOx. Following the encapsulation, the GOx catalyzes the oxidation of glucose and produces hydrogen peroxide, which is then used by HRP to oxidize ABTS. After measurement on our cAADS platform, a violin-scatter plot generated using data from 20,000 screened droplets shows a clear discrimination between the population with (2.300 ± 0.004 V) and without (2.280 ± 0.002 V) glucose that initiates the reaction (Figure S4).

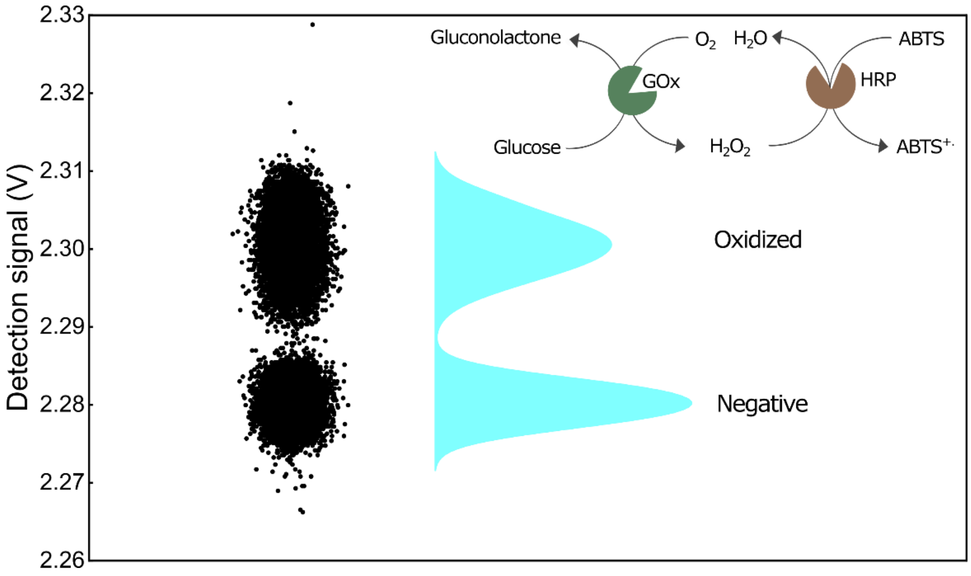

Figure S4. Violin-scatter plot generated from the signal measured using the cAADS platform.

S.6 Detection limit at 10 pL

We evaluated the detection limit of our setup using small 10-pL droplets by producing a population comprising 4 different ABTS concentrations (0 mM, 0.25 mM, 0.5 mM, and 1 mM). The signal from the droplets was measured by loading them into the cAADS module (Figure S2d). A histogram generated from 82,000 processed droplets shows the four expected peaks corresponding to each ABTS concentration (Figure S5). This histogram is then fit to a sum of four Gausssian components (green for 0 mM, orange for 0.25 mM, light blue for 0.5 mM, and deep blue for 1 mM; Figure S5) to determine the means and standard deviations of each concentration. The signal increases from 2.328 ± 0.002 V at 0 mM to 2.368 ± 0.003 V at 1 mM.

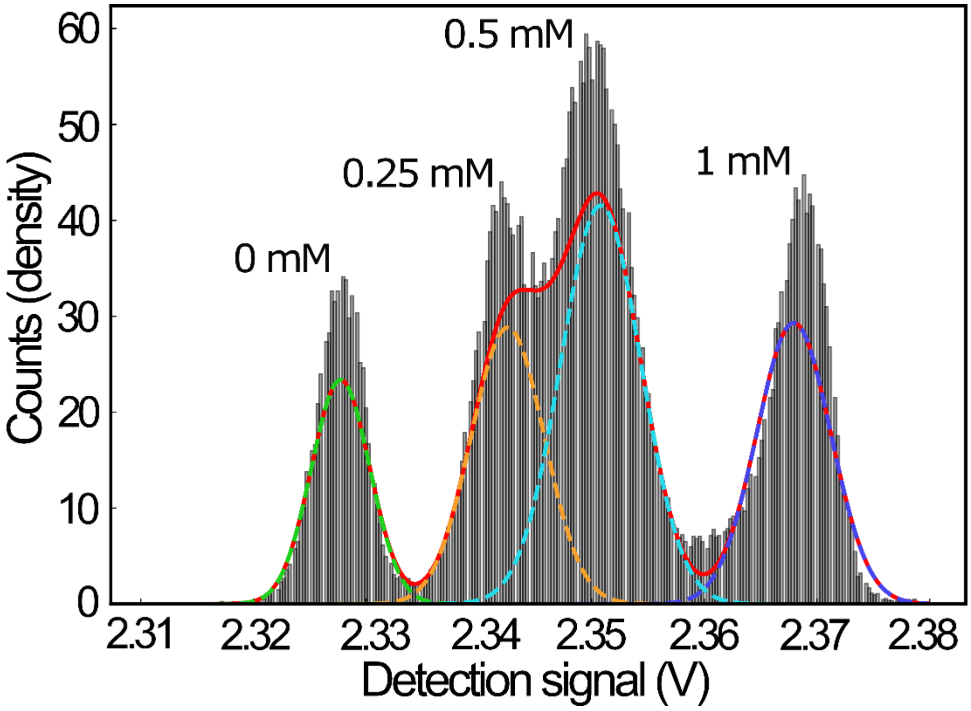

Figure S5. Histogram of the absorance signals from 82,000 droplets containing 0 mM, 0.25 mM, 0.5 mM, and 1 mM ABTS. The data is fit to a sum of four Gausssian subpopulations.

S.7 Experimental determination of sorting condition

Droplets loaded into the analysis module for optical detection and sorting have to be accurately spaced out to avoid impact at the junction and false positives.[5] The determination of the optimal pumping condition that guarantees even spacing of the droplets and the corresponding sorting efficiency is often carried out by optical inspection of the downstream of the channel. Nevertheless, imaging the downstream was challenging on our setup due to the small field of view (0.46 mm) of the objective lens. Thus, we used a fluorescence-activated droplet sorting (FADS) setup[6] with a lower magnification lens (Plan 40×, Olympus) allowing imaging of the downstream. Subsequently, the experimentally determined sorting condition (Table S2) was employed to conduct sorting on our cAADS setup. The first five sorting conditions listed in the table are for 50 pL droplets while the sixth belongs to 10 pL droplets. The cAADS module used to screen 10 pL droplets has an additional spacing-oil channel on the side that is used to further tune the gap between the droplets. Thus, the two flow rates shown in the Oil column correspond to the main and additional oil flow rates, respectively.

Table S2. Experimentally determined droplet sorting condition

No	Flow rate Q (μL/hr)		Average Sorting frequency (Hz)	Sorting condition		
	Oil	Droplets		Voltage (Vpp)	Cycle	Field frequency (kHz)
**1**	1000	75	203	1000	15	30
**2**	2400	200	507			
**3**	2800	350	1030		10	
**4**	5500	600	2182	1400	8	
**5**	7000	700	2471			
**6***	3300 / 3000	300	5300	1100	5	

S.8 Comparative performances analysis of cAADS

The screening and sorting performace of our platform is evaluated against previously reported absorbance-based high throughput droplet screening systems, based on minimum detectable droplet size and maximum achieved throughput (Figure S6). Gielen et al. reported a maximum sorting frequency of 300 Hz using 180 pL droplets.[7] A study by Medcalf et al. demonstrated an enhanced throughput of 1000 Hz with 50 pL droplet.[8] More recently, Jain et al. achieved 1500 Hz sorting at 85% efficiency with 50 pL droplets. [9] In contrast, our system attains a a maximum screening frequency of 5400 Hz with 10 pL droplets, and a maximum sorting frequency of 2.6 kHz with 50 pL droplets.

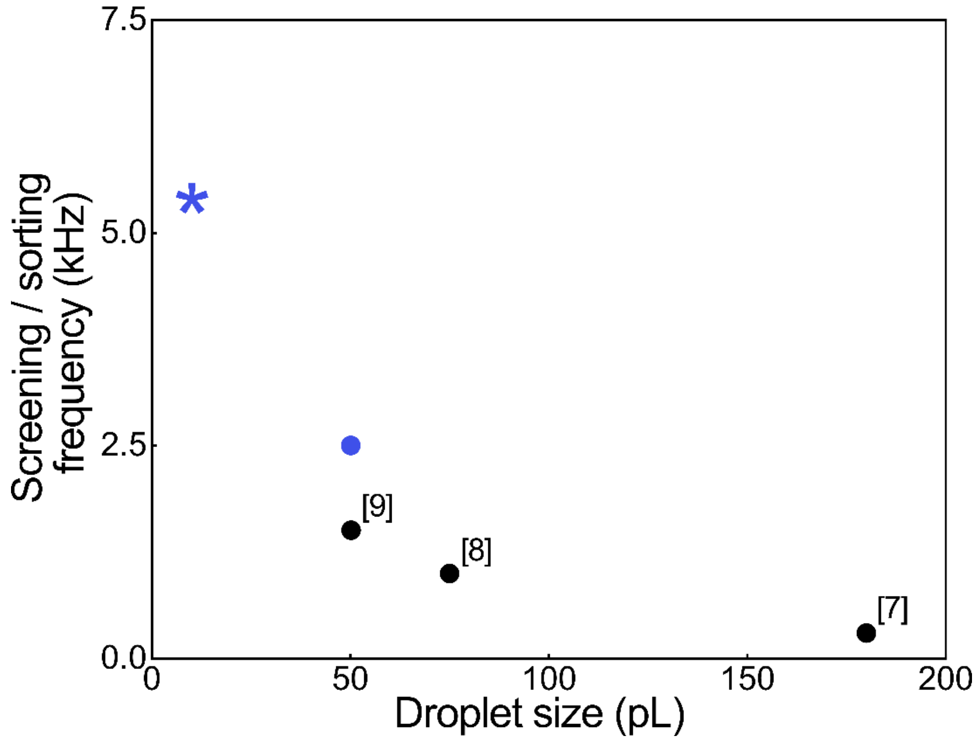

Figure S6. Screening (asterisk) and sorting (dot) performances of the newly developed cAADS (blue) compared to previously reported absorbanced-based droplet screening works.

S.9 Ultrahigh throughput fluorescence-activated droplet sorting (FADS)

The sorting conditions (i.e., high-voltage AC and oil/sample pumping flow rates) were first optimized on a FADS station with a lower magnification lens (Plan 20×, Olympus) which allowed post-sorting imaging of the cAADS module downstream. A population of 10 pL droplets with 10 mM oxidized ABTS were used for the experiment. The sorting was performed in "alternate" mode to ensure that an electrical signal is triggered for every other detected signal (i.e., 50% of the screened droplets are sorted; Figure S7). This strategy is put in place to mimic the actual sorting experiment that will be conducted on the cAADS platform where 50% of the droplet population will be active (contains the oxidizing BOD enzyme) and sorted. Figure S7a shows a screenshot of the LabVIEW user interface during sorting at 5478 Hz under the conditions determined experimentally (Table S2). A montage generated from a video (Movie S1) taken during sorting shows accurate "alternate" sorting (Figure S7b).

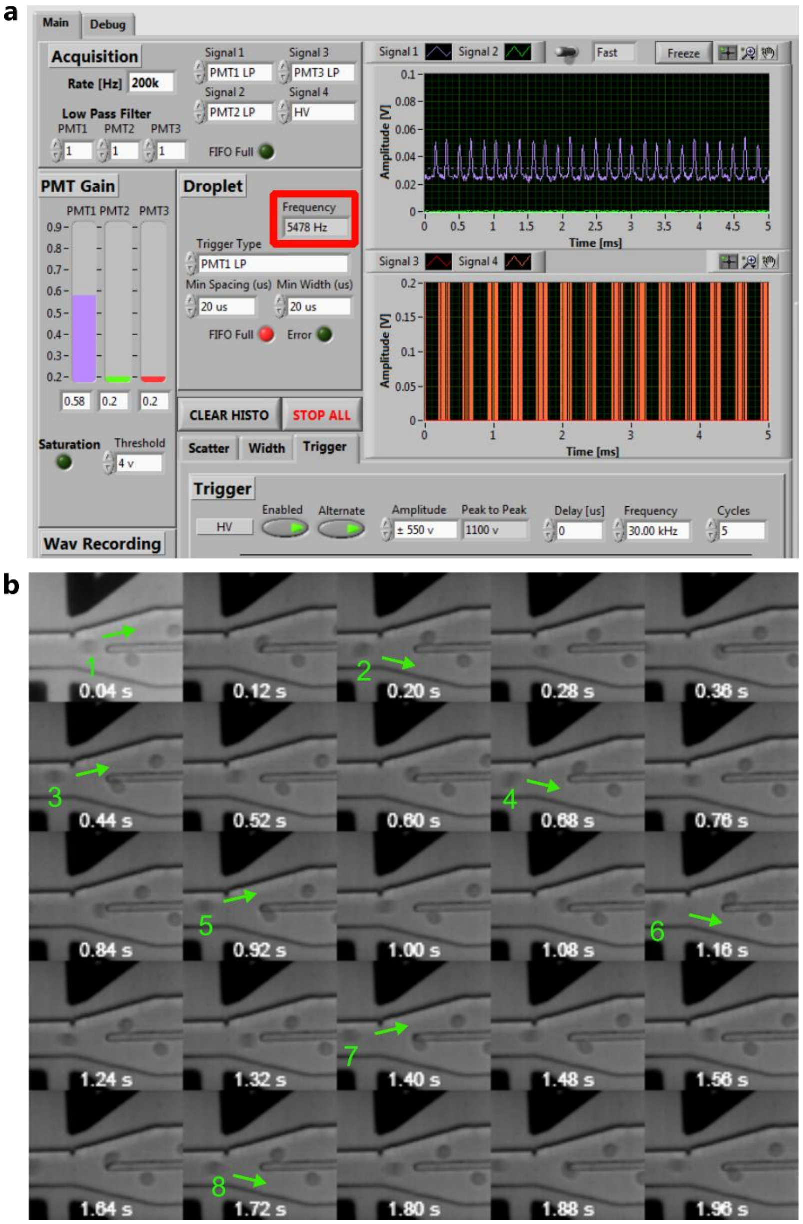

Figure S7. (a) A screenshot of the LabVIEW user interface during sorting shows a frequency of 5478 Hz and "alternate" trgger signals (red) corresponding to the detected droplet signals (purple peaks). Note: Peaks are inverted relative to the raw transmittance traces, so that higher-absorbance droplets yield higher detection voltages for easier sorting thresholding. (b) A montage made from a sequence of images extracted from a video taken during sorting and slowed down by 0.000735. The green numbers indicate a new droplet entering the field of view, and the arrows show whether the droplet is sorted (i.e., bottom channel) or passes to the top channel.

S.10 Ultrahigh-throughput screening on cAADS platform

To extend the limits of our ultrahigh-throughput screening capabilities, populations of monodisperse 10 pL droplets containing either both the enzyme BOD and the substrate ABTS or ABTS alone (negative control) were loaded into the cAADS module (Figure S2d). Although sorting was unsuccessful due to the absence of an optical system capable of imaging downstream of the module, making it difficult to precisely locate a detection zone that ensures accurate sorting, screening was nonetheless successfully performed at frequencies up to 5.4 kHz (Figure S8).

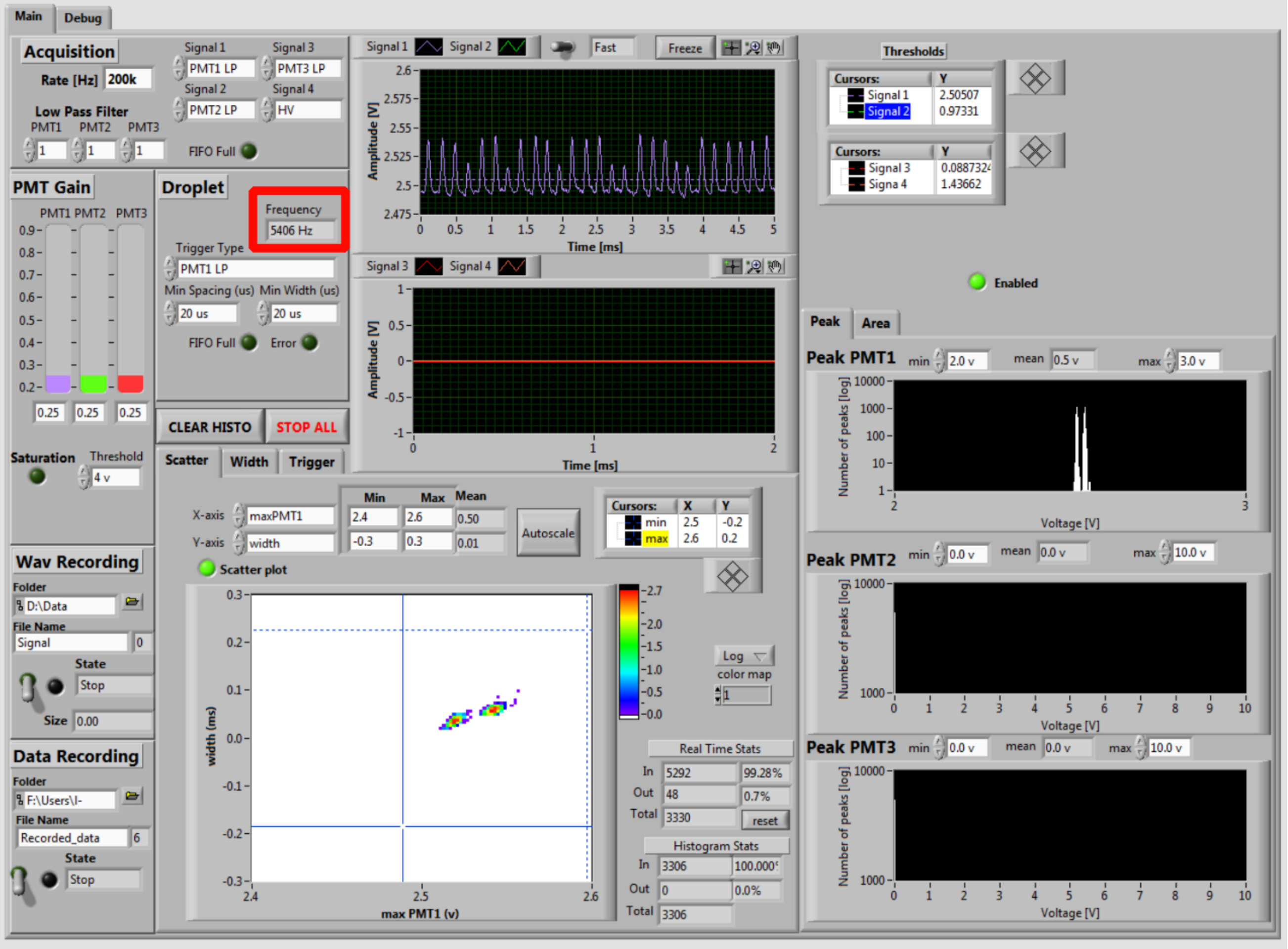

Figure S8. (a) A screenshot of the LabVIEW user interface during screening on the cAADS platform shows a frequency of 5406 Hz. A scatter plot embedded within the software which shows two distinct droplet populations validates the high discrimination accuracy of the system. Note: Peaks are inverted relative to the raw transmittance traces, so that higher-absorbance droplets yield higher detection voltages for easier sorting thresholding.

S.11 Enrichment of active BOD

To further demonstrate the performance of our platform, we conducted screening and enrichment of a target enzyme using 50 pL droplets. A bacterial strain that express an active wild-type or inactive mutant BOD variant was compartmentalized with ABTS. The distribution of bacterial cells followed Poisson's distribution.[10] Following off-chip incubation at 80 °C for 30 min, absorbance was measured by loading the droplets into the cAADS module (Fig S2e). Based on the cell distribution, we expect 4.50% active single-cell droplet population and 0.22% active population with two or more cells. The histograms in Figure S9 show the distribution of active and inactive populations derived from independent experimental runs. Including a similar histogram shown in Figure 4d (main MS), the average active population is calculated to be 4.46%. The slight variations could be due to random error during formulation or incomplete enzymatic reactions. Although the sorting frequency was conservatively kept below 2.30 kHz in most experiments, the system demonstrated accurate sorting at frequencies up to 2.60 KHz (Figure S9a, b).

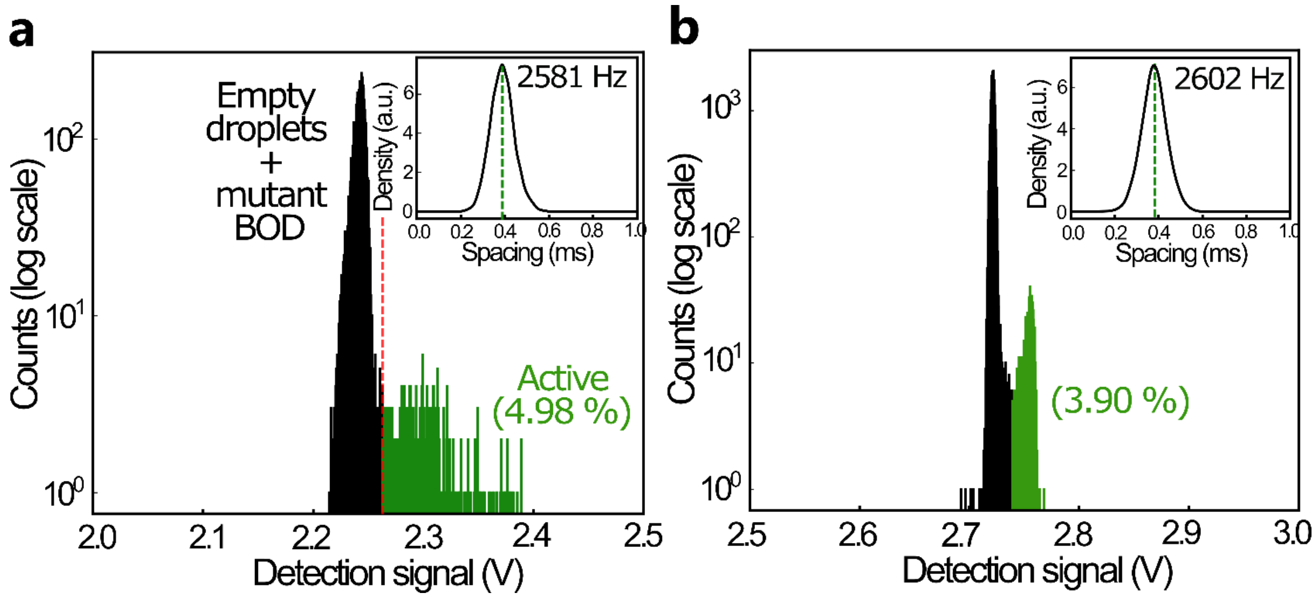

Figure S9. 1D histogram showing the signal from inactive/empty (black) and active (green) droplets after encapsulation (50 pL) and incubation (80 °C, 30 min) of BOD-expressing *E.coli* with ABTS. Droplets with a signal above the systematically placed threshold (red dotted line) are sorted into the positive channel and collected for subsequent analysis. The inset density plot shows the distribution of the spacing between each measurement (i.e., droplet) with a peak (green dotted line) at (a) 0.4 ms and (b) 0.384 ms corresponding to 2581 Hz and 2602 Hz, respectively.

S.12 Plasmid DNA recovery, concentration, amplification, and sequencing

The unsorted and sorted droplets (~25,000) were destabilized using 1H,1H,2H,2H perfluorooctanol (PFO, Alfa Aesar, USA) to recover the plasmids based on a previously reported protocol with few minor modifications.[11] Briefly, 150 µL PFO and 50 µL MQ water were added to a 1.5 mL low-DNA binding tube (Corning, USA) containing sorted droplets suspended in 300 µL HFE oil. The tube was then vortexed and centrifuged (16,000 g) for 1 min each. The upper aqueous layer containing the DNA was then carefully pipetted out and transferred to a new low DNA binding tube. Further recovery of the DNA was conducted by adding 100 µL PFO and 50 µL MQ water to the oil phase remaining in the original collection tube, followed by vortexing and subsequent centrifugation (16000 g) for 1 min each. The upper aqueous layer was pipetted out and added to the previously collected aqueous layer. The plasmid DNA suspended in the 100 µL aqueous layer was then purified and concentrated to a final elution volume of 5 µL using a DNA Clean & Concentrator Kit (Zymo Research, USA) according to the manufacturer's instructions, with some modifications as detailed in a previous report.[11] The open reading frame of the enzyme was amplified by two PCR runs with Q5 polymerase (NEB) and 26 cycles (30s at 96 °C for denaturation, 30s at 55 °C for annealing, and 60s at 72 °C for elongation). The first run used 1 µL of the above extractions as a template with primers 1 and 2 (Figure S10a). The second run was amplified from 1 µL of the first PCR with primers 3 and 4 (Figure S10a). The gel electrophoresis results in Figure S10b show the amplifications after the first and second runs. The final amplification is cleaned and concentrated with the same "DNA Clean and Concentrate" kit to a final volume of 10 µL. 5 µL of each sample is sent for sequencing with primer 5 (Figure S10a, Genwiz).

a

No	Primer sequence
1 (Forw.)	cgtagaggatcgagatctcgatccc
2 (Rev.)	cctcctttcagcaaaaaacccctc
3 (Forw.)	atgaacctagaaaaatttgttgacg
4 (Rev.)	ataatatccatcggcctcatc
5	gctcactcttactggtacacaggat

b

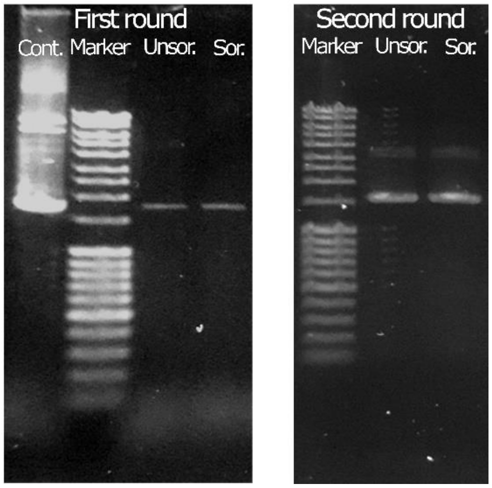

Figure S10. (a) A list of sequences for primers used during the the two successive amplifications and sequencing. (b) Gel electrophoresis results for the amplified DNA fragment.

References

[1] F. Durand, C. H. Kjaergaard, E. Suraniti, S. Gounel, R. G. Hadt, E. I. Solomon, N. Mano, *Biosensors and Bioelectronics* **2012**, *35*, 140–146.

[2] S. Gounel, J. Rouhana, C. Stines-Chaumeil, M. Cadet, N. Mano, *Journal of Biotechnology* **2016**, *230*, 19–25.

[3] R. Bennett, A. Rathore, S. Gounel, A. Lielpetere, T. M. B. Reichhart, K. Jayakumar, R. Ludwig, A. K. G. Felice, D. Leech, W. Schuhmann, A. Mount, N. Mano, C. Boiziau, *Advanced Sensor Research* **2024**, *3*, 2400056.

[4] T. Beneyton, F. Coldren, J.-C. Baret, A. D. Griffiths, V. Taly, *Analyst* **2014**, *139*, 3314–3323.

[5] J.-C. Baret, O. J. Miller, V. Taly, M. Ryckelynck, A. El-Harrak, L. Frenz, C. Rick, M. L. Samuels, J. B. Hutchison, J. J. Agresti, D. R. Link, D. A. Weitz, A. D. Griffiths, *Lab Chip* **2009**, *9*, 1850–1858.

[6] T. Beneyton, T. Rossignol, in *Yarrowia Lipolytica: Methods and Protocols* (Eds.: I. Wheeldon, M. Blenner), Springer US, New York, NY, **2021**, pp. 205–219.

[7] F. Gielen, R. Hours, S. Emond, M. Fischlechner, U. Schell, F. Hollfelder, *Proceedings of the National Academy of Sciences* **2016**, *113*, E7383–E7389.

[8] E. J. Medcalf, M. Gantz, T. S. Kaminski, F. Hollfelder, *Anal. Chem.* **2023**, *95*, 4597–4604.

[9] A. Jain, M. Teshima, T. Buryska, D. Romeis, M. Haslbeck, M. Döring, V. Sieber, S. Stavrakis, A. de Mello, *Angewandte Chemie International Edition* **2024**, *63*, e202409610.

[10] S. Köster, F. E. Angilè, H. Duan, J. J. Agresti, A. Wintner, C. Schmitz, A. C. Rowat, C. A. Merten, D. Pisignano, A. D. Griffiths, D. A. Weitz, *Lab Chip* **2008**, *8*, 1110–1115.

[11] J. D. Schnettler, O. J. Klein, T. S. Kaminski, P.-Y. Colin, F. Hollfelder, *J. Am. Chem. Soc.* **2023**, *145*, 1083–1096.
